# The effect of gender on clinical characteristics and diagnostic process of obstructive sleep apnea syndrome

**DOI:** 10.1007/s00405-026-10345-2

**Published:** 2026-06-10

**Authors:** Yildiz Ucar, Caner Incekas, Keziban Ucar Karabulut, Meltem Karacan Golen

**Affiliations:** 1 Department of Pulmonary Disease, Faculty of Medicine, Başkent University, Hocacihan mah. Saray caddesi No:1, Selcuklu, Konya, 42080 Turkey; 2 Department of Biostatistics, Faculty of Science, Baskent University, Ankara, Türkiye; 3 Department of Emergency Medicine, Baskent University, Ankara, Türkiye; 4 Department of Neurology, Konya Training and Research Hospital, Baskent University, Konya, Türkiye

**Keywords:** Obstructive sleep apnea syndrome, Gender differences, Hypoxemia, Inflammation, Polysomnography, Cardiopulmonary involvement

## Abstract

**Background:**

Obstructive sleep apnea syndrome (OSAS) has long been considered a predominantly male disorder; however, increasing evidence suggests substantial sex-related differences in clinical presentation and disease progression.

**Methods:**

This retrospective study included adult patients diagnosed with OSAS by polysomnography between January 2020 and January 2026. Demographic characteristics, polysomnographic findings, laboratory parameters, and echocardiographic measurements were analyzed. Systemic inflammation was assessed using the monocyte-to-HDL ratio, monocyte-to-LDL ratio, systemic immune-inflammation index (SII), systemic inflammatory response index (SIRI), and pan-immune-inflammation value (PIV).

**Results:**

A total of 230 patients were included (68.3% male, 31.7% female). Female patients were older and had higher body mass index at diagnosis (*p* < 0.001). Mean and minimum oxygen saturation levels were significantly lower in women (*p* < 0.01), who also exhibited higher pulmonary artery pressure and lower ejection fraction (*p* < 0.001). In contrast, monocyte-based inflammatory indices (MON/HDL, MON/LDL, and SII) were significantly higher in men. No significant sex-related differences were observed for SIRI or PIV.

**Conclusion:**

OSAS demonstrates distinct sex-specific clinical and inflammatory phenotypes. While women tend to present at a later stage with more pronounced cardiopulmonary involvement, men exhibit a greater systemic inflammatory burden. These findings support the importance of incorporating sex-based considerations into diagnostic and therapeutic strategies for OSAS.

## Introduction

Obstructive sleep apnea syndrome (OSAS) is a significant public health problem associated with cardiovascular, metabolic, and neurocognitive morbidity and mortality, characterized by recurrent episodes of upper airway collapse during sleep [1]. 

For many years, it was primarily considered a “male disease,” and epidemiological studies have shown a higher prevalence in men than in women [2, 3]. This perspective has led to significantly underrecognition and delayed diagnosis of OSAS in female patients [4, 5]. 

Recent large studies in this field have revealed that OSAS is more common in women than previously thought, but clinical presentation and polysomnographic features differ significantly according to gender. In women, OSAS may present with more non-specific complaints such as fatigue, insomnia, morning headache, depressive mood, anxiety, and difficulty concentrating, instead of the classic complaints of loud snoring and witnessed apnea [6, 7]. Possible mechanisms for gender-related differences in OSAS pathophysiology include upper airway anatomy, ventilatory control, fat distribution, and the effects of sex hormones [2, 5]. The increased risk of OSAS in the postmenopausal period supports the role of hormonal changes in disease development and clinical phenotype [5, 6]. Conversely, the more pronounced features such as visceral fat accumulation and increased neck circumference in men may be associated with a more typical clinical presentation and diagnostic referral [6–8]. 

In the diagnostic process, methods such as polysomnography (PSG) and/or home sleep apnea testing in appropriate patients are basic diagnostic tools; however, referral of the patient to these tests is often based on clinical suspicion [9]. Therefore, insufficient recognition of gender specific clinical cues and the differing performance of screening approaches in some populations may increase diagnostic disparities [6, 8]. In this context, a more detailed evaluation of the impact of gender on the clinical features and diagnostic process of OSAS is important for early diagnosis, appropriate referral, and the development of personalized management strategies [10]. This study aimed to investigate gender-based clinical and diagnostic differences in patients diagnosed with OSAS. 

## Materials and methods

This study was conducted as a single-center, retrospective, cross-sectional study including individuals aged 18 years and older who were diagnosed with OSAS using polysomnography (PSG) in our center’s sleep laboratory between January 2020 and January 2026. Patients under 18 years of age, those with incomplete or technically inadequate PSG records, and those with missing essential demographic or PSG parameters were excluded. Ethical committee approval and/or patient consent requirements were fulfilled in accordance with institutional regulations. Patient data were analyzed after anonymization. The study was conducted in accordance with the principles of the Declaration of Helsinki (2024 revision) and reported following the STROBE guidelines.

Demographic data, medical history, body mass index (BMI), and Epworth Sleepiness Scale (ESS) scores were obtained from hospital records. Laboratory parameters were retrospectively extracted from patients’ medical records as part of routine clinical evaluation and were not obtained as part of a standardized sleep laboratory protocol. All data were recorded and compiled in an Excel database for analysis.

## Data sources and data collection

Data were obtained retrospectively from the hospital information management system and sleep unit records. The following information was recorded by reviewing the files of patients diagnosed with OSAS: demographic data (age, gender, body mass index, comorbidities), polysomnography data (Apnea-Hypopnea Index (AHI), minimum SpO₂, mean SpO₂, sleep stage distribution, and total sleep duration), and laboratory data. Laboratory parameters were obtained from measurements performed within six months prior to polysomnography, and when multiple measurements were available, the most recent values were selected for analysis. These data were extracted retrospectively from hospital records as part of routine clinical evaluation and were not part of a standardized study protocol.

Echocardiographic parameters (pulmonary artery pressure and ejection fraction) were obtained retrospectively from hospital records as part of routine clinical evaluation and were not part of a standardized study protocol. Echocardiographic measurements were selected from examinations performed within six months prior to polysomnography, and when multiple measurements were available, the most recent values were used for analysis.

To evaluate the effect of gender on clinical characteristics and the diagnostic process, patients were grouped as female and male, and variables were compared between these groups.

### Polysomnography (PSG)

Overnight PSG was performed using the Philips Respironics Alice 5 Diagnostic Sleep System to record sleep stages and cardiorespiratory parameters. Monitoring included multichannel electroencephalography (C3, C4, O1, O2, Fp1, Fp2, F3, F4, P3, P4), submental electromyography, bilateral electrooculography, electrocardiography, assessment of oronasal airflow using both a thermal sensor and nasal pressure transducer, body position sensors, thoracic and abdominal respiratory effort belts based on inductance plethysmography, finger pulse oximetry for arterial oxygen saturation, bilateral leg electromyography for movement detection, and tracheal sound recording.

Apnea was defined as a reduction of airflow by ≥ 90% lasting at least 10 s, while hypopnea was defined as a ≥ 30% reduction in nasal pressure signal accompanied by a ≥ 3% oxygen desaturation or an arousal lasting at least 10 s. Disease severity was classified according to the AHI as mild (5–14.9 events/hour), moderate (15–29.9 events/hour), and severe (≥ 30 events/hour). An AHI below 5 events/hour in the presence of snoring was considered simple snoring.

### Ethical approval

The study protocol was approved by the Institutional Ethics Committee of Baskent University Institutional Review Board (Approval No: KA26/14, Date: 13/01/2026).

### Calculation of systemic inflammation markers

Blood cell counts were obtained retrospectively from the patient's medical records. Blood samples were obtained within six months prior to polysomnography, and the most recent measurements were used for analysis.

Laboratory parameters, Monocyte-to-LDL ratio (MLR), Monocyte-to-HDL ratio (MHR), PIV, SII and SIRI indices were compared. White Blood Cell (WBC), neutrophil, Monocyte, lymphocyte, platelet and hemoglobin levels were recorded. PIV, SII, SIRI, MLR, MHR were calculated according to the following formulas.

$$\;\begin{array}{c}\mathrm{PIV}=\mathrm{Neutrophil},\;\mathrm x103/\mathrm{uL}\;\times\mathrm{Platelet},\;\mathrm x103/\mathrm{uL}\\\times\mathrm{Monocyte}\;\mathrm x103/\mathrm{uL}/\mathrm{lymphocyte}\;\mathrm x103/\mathrm{uL}\end{array}$$

$$\begin{array}{c}\mathrm{SII}\;=\;\mathrm{Neutrophil},\;\mathrm x103/\mathrm{uL}\\\times\;\mathrm{Platelet},\;\mathrm x103/\mathrm{uL}\;/\;\mathrm{lymphocyte}\;\mathrm x103/\mathrm{uL}\end{array}$$

$$\begin{array}{c}\mathrm{SIRI}\;=\;\mathrm{Neutrophil},\;\mathrm x103/\mathrm{uL}\\\times\;\mathrm{Monocyte}\;\mathrm x103/\mathrm{uL}\;/\;\mathrm{lymphocyte}\;\mathrm x103/\mathrm{uL}\end{array}$$

$$\mathrm{MLR}=\;\mathrm{Monocyte}\;\mathrm x103/\mathrm{uL}\;/\;\mathrm{LDL}\;\mathrm{cholesterol}\;(\mathrm{mg}/\mathrm{dL})$$

$$\mathrm{MHR}\;=\;\mathrm{Monocyte}\;\mathrm{count}\;(\times10^9/\mathrm L)\;/\;\mathrm{HDL}\;\mathrm{cholesterol}\;(\mathrm{mg}/\mathrm{dL})$$

### Statistical analysis

Statistical analysis of the data was performed using the IBM SPSS Statistics (Version 25.0, IBM Corp., Armonk, NY, USA) software package. The normality of the variables was examined using visual (histograms and probability plots) and analytical methods (Kolmogorov-Smirnov). Descriptive statistics were presented as median (minimum-maximum) values for quantitative data that did not show a normal distribution, and additionally as mean ± standard deviation (Mean ± SD). Categorical variables (if any) were expressed as number (n) and percentage (%). For comparisons of quantitative variables between male and female groups, the non-parametric Mann-Whitney U test was used because the data did not satisfy the assumption of normal distribution. For intergroup comparisons of categorical variables, the Pearson Chi-Square test (or Fisher's Exact Test where appropriate) was used. Logistic regression analysis was applied to determine the factors affecting female gender (dependent variable: Male=0, Female=1). In the first stage, a univariate (binary) logistic regression analysis was performed for each independent variable. In the second stage, variables that showed significance at the p<0.10 level in the univariate analysis and/or were considered to have clinical importance were included in the multivariate (multiple) logistic regression model using the forward (LR) method to identify independent determinants. Age and BMI were also evaluated as potential confounding variables during model construction.The results are reported with the regression coefficient (B), adjusted odds ratio (aOR), 95% confidence interval (CI), and p-value. Model fit was evaluated with the Hosmer-Lemeshow test. In all statistical analyses, a p-value <0.05 was considered statistically significant.

## Results

A total of 230 patients were included in the study, of whom 68.3% (n=157) were male and 31.7% (n=73) were female. The mean age of the study population was 50.16±12.82 years. The mean apnea–hypopnea index (AHI) indicated a moderate-to-severe OSAS population. Mean oxygen saturation was 89.15±5.22%, and the lowest oxygen saturation was 77.73±11.18%. Hematological and biochemical parameters, as well as inflammatory indices, are presented in detail in Table 1.

**Table 1:** Descriptive statistics for the variables

	Mean	Standard deviation
Age	50,16 ± 12,82	49 (19–80)
AHI	36,27 ± 25,33	29,8 (2,2-102,7)
Total sleep activity	79,34 ± 17	84,7 (7,3–99,9)
Sleep lat	13,53 ± 18,56	7 (0-101,1)
REM AHI	32,48 ± 23,01	30,3 (0-100)
NREM AHI	36,25 ± 24,34	31,25 (2-102)
SUPIN AHI	45,34 ± 27,22	43,8 (0-114,9)
Mean SPO2	89,15 ± 5,22	90 (64–95)
Lowest SPO2	77,73 ± 11,18	81 (40–96)
EF%	59,13 ± 36,17	60 (20–600)
PAP	23,63 ± 7,17	20 (20–65)
Hemoglobin	14,68 ± 1,74	14,5 (7,1–18,8)
Neutrophil	4,41 ± 1,64	4,11 (2,15 − 12,5)
Monocyte	0,62±,23	0,59 (0,09 − 1,55)
Lymphocyte	2,63 ± 1,4	2,5 (0,07–19,8)
Platelet	240,53 ± 64,06	238 (0,34–516)
Eosinophil	0,21±,48	0,15 (0–7,16)
Baso	1,45 ± 20,48	0,08 (0-310)
Uric acid	5,85 ± 1,10	5,6 (0,12 − 9,2)
LDL	126,94 ± 32,88	125 (8,4-207)
HDL	48,57 ± 10,86	47 (25–123)
Triglyceride	168,63 ± 76,77	160 (2,3-606)
Albumin	5,27 ± 9,81	4,5 (3,4-153)
Total Cholesterol	183,18 ± 48,10	180 (1-306)
BMI	36,14 ± 24,72	32,45 (21,4-275)
MHR	0,01±,01	0,01 (0–0,03)
MLR	0,01±,00	0 (0–0,06)
SII	581,66 ± 1761,04	391,77 (0,73-26460)
SIRI	1,35 ± 2,28	0,96 (0,03–29,31)
PIV	330,23 ± 697,96	222,3 (0,56-10054,8)

*AHI* Apnea Hypopnea Index, *BMI* Basal Mass Index, *PAP* Pulmonary Artery Pressure, *EF* Ejection Fraction, *LDL* Low-density lipoprotein, *HDL* High-density lipoprotein, *MH*: Monocyte/HDL, *MLR* Monocyte/LDL, *SII* Systemic Immune-Inflammation Index, *PIV* Pan-immune inflammation value, *SIRI* Systemic Inflammatory Response Index

**Fig. 1:**
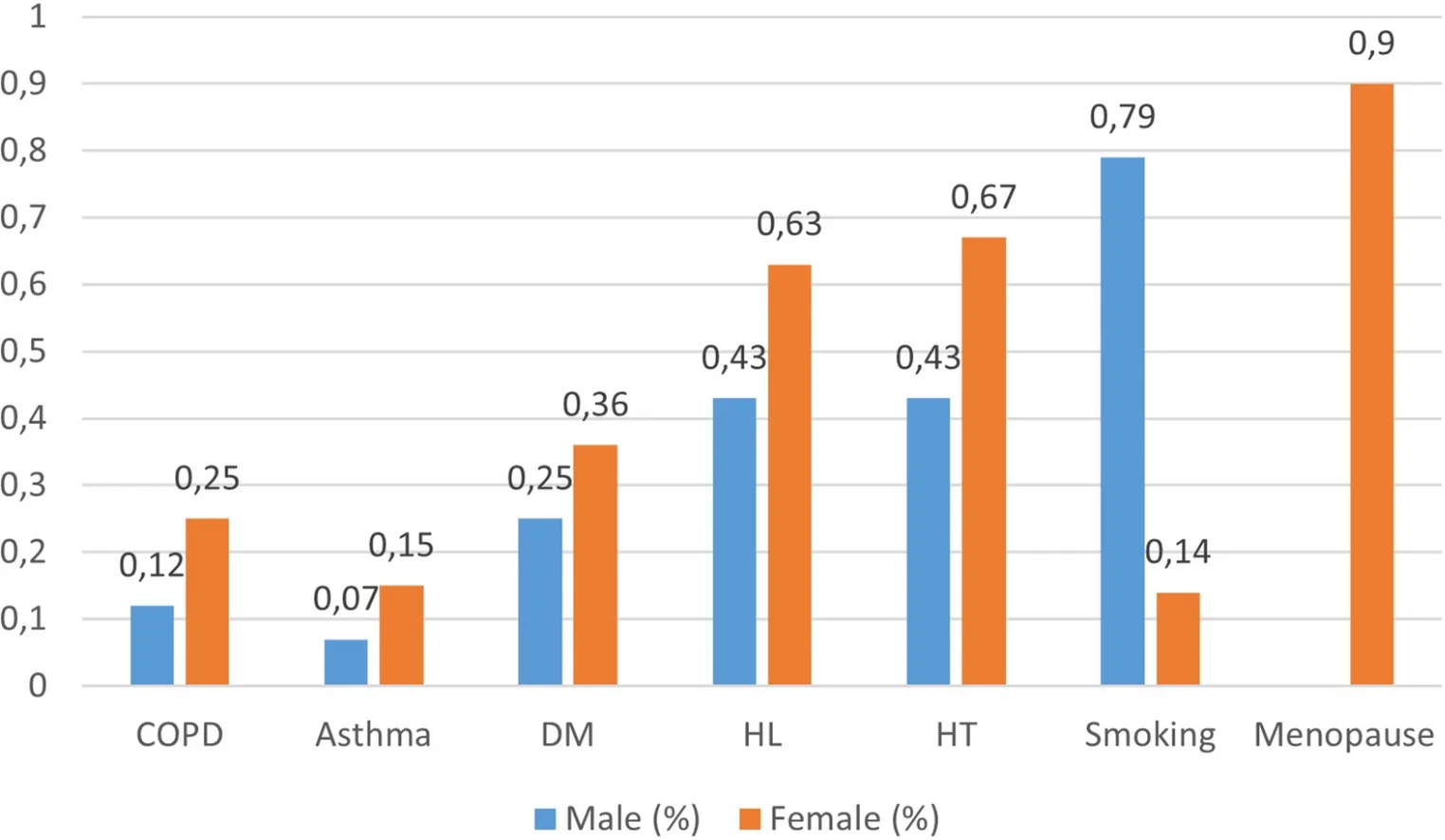
Sex-based distribution of comorbidities and menopausal status in patients with obstructive sleep apnea syndrome

Comorbidities were common in the cohort. Hypertension and hyperlipidemia were present in approximately half of the patients, and smoking was observed in more than half of the study population. The majority of female patients were postmenopausal (90.4%). Detailed clinical characteristics are shown in Table 2 and Fig. 1.

**Table 2:** Demographics of patients with OSAS

		*n*	%
Gender	Male	157	(68,26)
	Female	73	(31,74)
Snore	No	23	(10)
	Yes	207	(90)
COPD-Asthma	No	173	(74,24)
	Yes	57	(16,16)
Diabetes mellitus (DM)	No	164	(71,3)
	Yes	66	(28,7)
Hyperlipidemia	No	116	(50,43)
	Yes	114	(49,57)
Hypertension (HT)	No	114	(49,57)
	Yes	116	(50,43)
Atherosclerotic Heart Disease (ASHD)	No	193	(83,91)
	Yes	37	(16,09)
Atrial fibrillation (AF)	No	215	(93,48)
	Yes	15	(6,52)
Chronic Kidney Failure (CKF)	No	219	(95,22)
	Yes	11	(4,78)
Congestive Heart Failure (CHF)	No	203	(88,26)
	Yes	27	(11,74)
Smoking	No	96	(41,74)
	Yes	134	(58,26)
Menopause (Woman)	No	0	
	Yes	66	(90,4)

When comparisons were performed according to sex (Table 3), female patients were significantly older than male patients (median: 57 vs. 44 years, p < 0.001) and had higher BMI values (median: 36.2 vs. 31.3 kg/m², p < 0.001).

**Table 3:** Demographic, polysomnographic, and laboratory characteristics by gender

	Male		Female		*p*
	Mean ± SD	Median(Min.-Maks.)	Mean ± SD	Median(Min.Max.)	
Age	46,54 ± 11,58	44 (19–80)	57,93 ± 11,93	57 (26–80)	**< 0**,001*
AHI	34,72 ± 25,43	26 (3,8-100,7)	39,62 ± 24,94	34,8 (2,2-102,7)	0,081
Total sleep activity	79,79 ± 18,02	86,5 (7,3–99,9)	78,38 ± 14,63	78,7 (39,6–99,5)	0,151
Sleep lat	12,78 ± 18,36	6 (0–99)	15,15 ± 19,03	9 (0-101,1)	0,16
REM AHI	31,45 ± 22,92	29,3 (0–93,3)	34,69 ± 23,20	31,6 (0-100)	0,337
NREM AHI	37,98 ± 25,75	30,1 (2-101,8)	32,53 ± 20,66	33,8 (2-102)	0,287
SUPIN AHI	47,09 ± 26,8	37,4 (0-114,9)	41,57 ± 27,91	45,6 (4-113,6)	0,147
Mean SPO2	89,9 ± 4,23	91 (71–95)	87,53 ± 6,63	89 (64–95)	**0**,007*
Lowest SPO2	79,59 ± 9,52	82 (45–96)	73,73 ± 13,32	78 (40–92)	**0**,001*
EF%	61,11 ± 43,44	60 (40–600)	54,86 ± 6,77	55 (20–60)	**< 0**,001*
PAP	22,68 ± 6,22	20 (20–65)	25,68 ± 8,55	20 (20–60)	**< 0**,001*
Hemoglobin	33,11 ± 20,02	31,3 (21,4-275)	42,65 ± 31,81	36,2 (22,7-231)	**< 0**,001*
Neutrophil	15,21 ± 1,68	15,4 (7,1–18,8)	13,56 ± 1,26	13,6 (9,9–17,5)	**< 0**,001*
Monocyte	4,4 ± 1,61	4,17 (2,3–12,5)	4,42 ± 1,71	3,96 (2,15 − 9,88)	0,918
Lymphocyte	0,54±,23	0,53 (0,09 − 1,32)	0,66±,23	0,62 (0,12 − 1,55)	**< 0**,001*
Platelet	2,61±,83	2,53 (0,07 − 5,01)	2,67 ± 2,18	2,44 (0,53 − 19,8)	0,156
Eosinophil	230,09 ± 59,78	230 (0,34–389)	262,97 ± 67,52	253 (104–516)	**0**,001*
Basophil	0,24±,57	0,15 (0,01–7,16	0,16±,12	0,14 (0–0,47)	0,082
Uric acid	2,08 ± 24,73	0,09 (0-310)	0,09±,07	0,07 (0–0,47)	0,098
LDL	6,01±,97	6 (3,1–9,2)	5,51 ± 1,28	5,5 (0,12 − 8,8)	**0**,001*
HDL	127,25 ± 31	125 (29–200)	126,27 ± 36,82	125 (8,4-207)	0,749
Triglyceride	47,25 ± 8,96	47 (25–65)	51,41 ± 13,75	47 (27–123)	0,188
Albumin	173,92 ± 85,01	160 (2,3-606)	157,26 ± 53,82	163 (40–342)	0,486
Total Cholesterol	4,74±,71	4,5 (3,4–7)	6,41 ± 17,40	4,2 (3,8-153)	**< 0**,001*
BMI	182,5 ± 45,67	180 (2,71–306)	184,63 ± 53,26	182 (1-304)	0,449
MHR	0,01±,01	0,01 (0–0,03)	0,01±,01	0,01 (0–0,03)	**< 0**,001*
MLR	0,01±,00	0,01 (0–0,02)	0,01±,01	0 (0–0,06)	**0**,002*
SII	590,65 ± 2097,87	350 (0,73-26460)	562,08 ± 537,10	448,25 (32,22-4085,53)	**0**,004*
SIRI	1,47 ± 2,68	1 (0,1–29,31)	1,1±,90	0,9 (0,03–5,68)	0,065
PIV	352,3 ± 831,27	221,51 (0,56-10054,8)	282,09 ± 206,53	224,01 (7,41-1266,51)	0,790

*p < 0,05

In terms of respiratory and cardiac parameters, despite similar AHI values between groups, women demonstrated significantly lower mean and minimum SpO₂ levels (p=0.007 and p=0.001, respectively). In addition, pulmonary artery pressure (PAP) was significantly higher, while ejection fraction (EF%) was significantly lower in women compared to men (p < 0.001 for both).

**Fig. 2:**
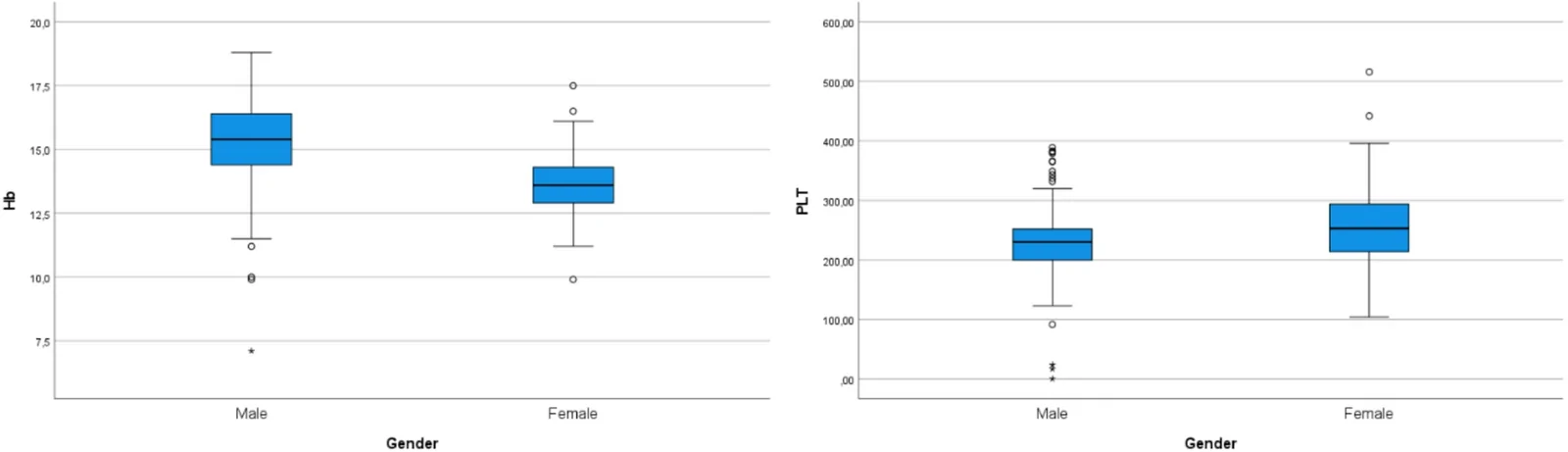
Distribution of hemoglobin (Hb) levels and platelet (PLT) counts according to sex in patients with obstructive sleep apnea syndrome

Regarding laboratory findings, hemoglobin, monocyte count, uric acid, and albumin levels were significantly higher in men, whereas platelet counts were higher in women (Fig. 2). No significant differences were observed between sexes in lipid profile parameters. Among inflammatory markers, monocyte-based indices (MON/HDL and MON/LDL) and SII were significantly higher in men (Table 3).

The distribution of comorbidities differed significantly between sexes (Table 4). Hypertension, hyperlipidemia, and COPD/asthma were more frequent in women, whereas smoking was markedly more prevalent in men (p < 0.001).

In univariate analyses, age, hypertension, hyperlipidemia, menopausal status, smoking, oxygen saturation parameters, cardiac parameters (EF% and PAP), and several laboratory variables were significantly associated with female sex.

**Table 4:** Comparison of symptoms and comorbidities by gender in patients with OSAS

		Gender				*p*
		Male		Female		
		*n*	%	*n*	%	
Snore	0	14	(8,92)	9	(12,33)	0,422
	1	143	(91,08)	64	(87,67)	
COPD-Asthma	0	126	(80,77)^b^	44	(60,27)^a^	**0**,**004**
	1	19	(12,18)^b^	18	(24,66)^a^	
	2	11	(7,05)	11	(15,07)	
Diabetes mellitus (DM)	0	117	(74,52)	47	(64,38)	0,114
	1	40	(25,48)	26	(35,62)	
Hyperlipidemia(HL)	0	89	(56,69)	27	(36,99)	**0**,**005**
	1	68	(43,31)	46	(63,01)	
Hypertansion (HT)	0	90	(57,32)	24	(32,88)	**< 0**,**001**
	1	67	(42,68)	49	(67,12)	
Atherosclerotic Heart Disease (ASHD)	0	129	(82,17)	64	(87,67)	0,290
	1	28	(17,83)	9	(12,33)	
Atrial fibrillation (AF)	0	147	(93,63)	68	(93,15)	1,000
	1	10	(6,37)	5	(6,85)	
Chronic Kidney Failure (CKF)	0	147	(93,63)	72	(98,63)	0,181
	1	10	(6,37)	1	(1,37)	
Congestive Heart Failure (CHF)	0	139	(88,54)	64	(87,67)	0,850
	1	18	(11,46)	9	(12,33)	
Smmoking	0	33	(21,02)	63	(86,30)	**< 0**,**001**
	1	124	(78,98)	10	(13,70)	
Menopause (Female)	0	7	(9,59)			**< 0**,**001**
	1	66	(90,41)			

0.no 1.yes

In the multivariate logistic regression model (Table 5), menopausal status, non-smoking, and the presence of COPD/asthma emerged as the strongest independent predictors of female sex. Several laboratory and physiological variables also remained independently associated with female sex. Detailed regression results are presented in Table 5. The model demonstrated high explanatory power (Nagelkerke R² = 0.787) and good classification performance.

**Table 5:** Univariate and multivariate logistic regression analysis of factors associated with gender (man: 0, woman=1)

	Binary logistic regression				Multiple logistic regression		
Variables	B	aOR (95% CI)**		*p*-değeri	B	aOR (95% CI)**	*p*-value
Age	0,08	1,083	(1,054 − 1,112)	**< 0**,**001**			
AHI	0,008	1,008	(0,997–1,019)	0,172			
Total sleep activity	-0,005	0,995	(0,979–1,011)	0,557			
Sleep lat.	0,007	1,007	(0,992–1,021)	0,368			
SUPIN AHI	-0,008	0,992	(0,982–1,003)	0,153			
Mean SPO2	-0,084	0,920	(0,871–0,971)	**0**,**002**	-0,198	0,821(0,729-0,923)	**0**,001*
Lowest SPO2	-0,046	0,955	(0,932–0,98)	**< 0**,**001**			
EF%	-0,12	0,887	(0,829–0,948)	**< 0**,**001**			
PAP	0,056	1,058	(1,016 − 1,102)	**0**,**007**			
Hemoglobin	-0,686	0,504	(0,401–0,632)	**< 0**,**001**	-0,823	0,439(0,297-0,649)	**< 0**,001*
Monocyte	-2,436	0,087	(0,02 − 0,374)	**0**,**001**			
Lymphocyte	0,031	1,031	(0,851–1,249)	0,753			
Platelet	0,009	1,009	(1,004 − 1,014)	**0**,**001**	0,012	1,012(1,002 − 1,022)	**0**,023*
Eosinophil	-1,879	0,153	(0,015 − 1,571)	0,114			
Basophil	-1,803	0,165	(0,003–10,832)	0,399			
Uric acid	-0,458	0,633	(0,471–0,849)	**0**,**002**	-0,83	0,436(0,236-0,807)	**0**,008*
HDL	0,036	1,037	(1,008 − 1,067)	**0**,**012**	0,086	1,090(1,015 − 1,17)	**0**,019*
Albumin	0,018	1,019	(0,979–1,06)	0,366			
BMI	0,026	1,026	(0,994–1,059)	0,118			
MHR	-124,666	0,000	(0–0)	**< 0**,**001**			
MLR	-12,749	0,000	(0–5,0347E25)	0,728			
SII	0,000	1,000	(1–1)	0,909			
SIRI	-0,158	0,854	(0,63 − 1,156)	0,306			
COPD-Asthma				**0**,**005**			**0**,010*
COPD-Asthma	0,998	2,713	(1,307-5,631)	**0**,**007**	1,669	5,308(1,125 − 25,049)	,035
COPD-Asthma(2)	1,052	2,864	(1,160-7,068)	**0**,**022**	2,477	11,907(2,012–70,449)	,006
DM	0,481	1,618	(0,889–2,944)	0,115			
HL	0,802	2,230	(1,26 − 3,945)	**0**,**006**			
HT	1,009	2,743	(1,533–4,907)	**0**,**001**			
Chronic Kidney Failure (CKF)	-1,589	0,204	(0,026 − 1,626)	0,133			
Smoking	-3,164	0,042	(0,02 − 0,091)	**< 0**,**001**	-4,423	0,012(0,003 − 0,057)	**< 0**,001*
Menopause (Woman)	2,231	9,309	(4,021–21,554)	**< 0**,**001**	3,144	23,192(4,704 − 114,343)	**< 0**,001*

*p < 0,05

## Discussion

Clinical studies investigating gender differences in obstructive sleep apnea syndrome (OSAS) generally report a higher prevalence among men, with a male-to-female ratio of approximately 3–5:1 [7]. In the adult population of the United States, the prevalence of OSAS has been estimated at approximately 39.1% in men and 26.0% in women, indicating that men have nearly 1.5 times higher prevalence [11]. In our study, the male-to-female ratio was 2.15:1. This finding is consistent with community-based studies reporting ratios close to 2:1, although it is lower than the stronger male predominance reported in some sleep laboratory cohorts. One possible explanation is the well-documented underdiagnosis of OSAS in women, which has been attributed to atypical clinical presentations and lower referral rates.

An important observation in our study was that women were diagnosed with OSAS at an older age than men. The findings regarding the distribution of OSAS by age and gender strongly support the older age at diagnosis observed in female patients in our study. Previous studies have shown that women with OSAS are frequently diagnosed 5–10 years later than men and often present with additional comorbidities such as obesity, hypertension, and endocrine disorders [12–15]. The higher age at diagnosis observed in women may be explained by hormonal and physiological factors. The decline in estrogen and progesterone levels after menopause may reduce upper airway stability and alter ventilatory control, thereby increasing the risk of OSAS later in life [16, 17]. Consistent with this mechanism, the majority of women in our cohort were postmenopausal.

BMI is another well-established determinant of OSAS. Previous studies have shown that although obesity increases the risk of OSAS in both sexes, women often require higher BMI levels to develop disease of comparable severity [15, 18]. In our study, BMI values were also significantly higher in women. Therefore, the greater cardiopulmonary involvement observed in women should be interpreted cautiously, as age and BMI differences between groups may have partially contributed to these findings. It should be considered that female patients in our cohort were both older and had higher BMI values, which may partially contribute to some of the observed differences in hypoxemia and cardiopulmonary findings between sexes.

Despite similar AHI values between men and women in our study, female patients demonstrated lower mean and minimum oxygen saturation levels. This finding suggests that the hypoxemic burden may be more pronounced in women even when OSAS severity appears comparable based on AHI alone. One possible explanation is that delayed diagnosis in women may lead to longer exposure to intermittent hypoxemia.

Intermittent hypoxemia is known to contribute to cardiovascular alterations through sympathetic activation, intrathoracic pressure changes, and endothelial dysfunction. These mechanisms may result in increased PAP and impairment of cardiac function. There are few studies that directly and thoroughly examine the relationship between OSAS and pulmonary hypertension (PH/PAP) based on gender; current evidence regarding sex differences in pulmonary hypertension among patients with OSAS is limited and sometimes inconsistent. Some studies suggest that female sex may be associated with a higher risk of pulmonary hypertension in OSAS cohorts, whereas other studies have reported a higher prevalence of OSAS among male patients with pulmonary hypertension [19–22]. In our cohort, PAP was significantly higher and ejection fraction was lower in female patients, suggesting a greater degree of cardiopulmonary involvement.

OSAS is also increasingly recognized as a systemic inflammatory condition. Various inflammatory markers such as CRP, ESR, and neutrophil-derived indices have been associated with disease severity [23–26]. However, data on sex-specific inflammatory profiles in OSAS remain limited. In our study, hemoglobin and monocyte levels were higher in men, whereas platelet counts were higher in women. These findings may reflect inherent sex-related hematological differences rather than disease-specific mechanisms [27–29]. It should also be noted that leukocyte-derived inflammatory indices may be influenced by inherent hematological differences between sexes. In addition, the substantially higher prevalence of smoking among male participants in our cohort may have contributed to the higher inflammatory indices observed in men.

Numerous publications examine the monocyte/HDL (MHR) ratio in detail for OSAS and show a strong relationship with severity. One study concluded that MHR could be used as a novel predictor for OSAS [30]. Similarly, monocyte-based inflammatory indices such as the MHR MLR were higher in male patients. Previous studies have reported that MHR may be associated with OSAS severity and cardiovascular risk [31, 32]. However, sex-based comparisons remain scarce. Our findings suggest that systemic inflammatory responses associated with OSAS may differ between men and women. It should also be noted that leukocyte-derived inflammatory indices may be influenced by baseline hematological differences between sexes. Additionally, the substantially higher prevalence of smoking among male participants in our cohort may have contributed to the higher inflammatory indices observed in men.

Although monocyte and lipid parameters have been frequently evaluated in OSAS research, studies examining the monocyte-to-LDL ratio remain limited. One study investigated the relationship between OSAS severity and MLR [33]. In our literature review, we did not identify studies that comparatively evaluated MLR according to sex in OSAS populations. Therefore, our findings provide additional insight into potential sex-related differences in monocyte-based inflammatory responses. To our knowledge, this is one of the few studies that simultaneously evaluates multiple monocyte-based and composite inflammatory indices (including MHR, MLR, SII, SIRI, and PIV) in OSAS patients with a specific focus on sex-related differences.

In recent years, novel composite inflammatory indices derived from complete blood count parameters, such as SII, SIRI, and PIV, have been increasingly studied as markers of systemic inflammation in OSAS [33–36]. These indices incorporate multiple immune cell types and may better reflect the inflammatory burden associated with chronic intermittent hypoxemia. In our study, SII was significantly higher in men, whereas SIRI and PIV did not differ significantly between sexes. These findings suggest that certain components of the inflammatory response may exhibit sex-related variation, while others remain relatively similar.

Overall, our findings support the concept that OSAS may present with sex-specific clinical and biological characteristics. Women tended to be diagnosed at older ages and demonstrated greater cardiopulmonary involvement, whereas men exhibited higher levels of certain inflammatory markers. These differences may reflect distinct biological phenotypes of OSAS and highlight the importance of considering sex-related factors in the diagnosis and clinical evaluation of patients with suspected sleep apnea. These findings may contribute to a better understanding of sex-specific phenotypes of OSAS and may support the development of more individualized diagnostic and therapeutic approaches in clinical practice. These findings may also encourage clinicians to consider sex-related differences when evaluating inflammatory markers and cardiopulmonary involvement in patients with suspected OSAS.

## Limitations

This study has several limitations that should be considered. First, its retrospective and single-center design may limit the generalizability of the findings. The study population consisted of patients referred to a tertiary sleep laboratory, representing a selected clinical population rather than the general community. Referral patterns, physician suspicion, and access to sleep testing may differ between men and women; therefore, the observed sex-related differences may partially reflect referral and selection bias rather than purely biological differences. Accordingly, the findings should be interpreted as characteristics of a sleep laboratory population rather than the natural epidemiology of OSAS in the general population.

Second, although a relatively large cohort of OSAS patients was included, causal relationships between inflammatory biomarkers and disease severity or cardiopulmonary outcomes cannot be established due to the observational design of the study. In addition, laboratory parameters were obtained at a single time point and may not fully reflect dynamic inflammatory changes over time. Although a defined time window was applied for echocardiographic data, the retrospective nature of data collection and the lack of a standardized acquisition protocol may have introduced variability in these measurements. 

Third, several potential confounding factors including medication use, hormonal status beyond menopausal classification, nutritional status, and unmeasured inflammatory conditions could not be fully controlled. Although patients with overt inflammatory and hematological disorders were excluded as much as possible, subclinical conditions may still have influenced biomarker levels.

Finally, while sex-based comparisons were the primary focus of this study, subgroup analyses according to OSAS severity or menopausal status were limited by sample size considerations. Further analyses according to OSAS severity categories may provide additional insight into whether the observed inflammatory differences are related to sex or disease severity, and this should be explored in future studies with larger cohorts. Moreover, although novel inflammatory indices such as SII, SIRI, PIV, MHR, and monocyte/LDL ratios were evaluated, standardized threshold values for OSAS populations have not yet been clearly established, which may limit their clinical interpretability.

## Conclusion

This study reveals that obstructive sleep apnea syndrome can present with different clinical and biological phenotypes in female and male patients. Female OSAS patients were diagnosed at an older age and with a higher body mass index; they exhibited more pronounced cardiopulmonary involvement with lower oxygen saturation, higher pulmonary artery pressure, and lower ejection fraction. These findings suggest that OSAS is often diagnosed late in women and that the disease is often clinically recognized after advanced physiological involvement has developed. On the other hand, the higher monocyte-based inflammatory indices (MON/HDL, MON/LDL, and SII) found in male patients suggest that the systemic inflammatory burden accompanying OSAS may be more dominant in men. Evaluation of next-generation inflammatory biomarkers (SII, SIRI, and PIV) supports the idea that OSAS is not only a mechanical respiratory disorder but also a systemic inflammatory disease. In conclusion, it can be said that OSAS exhibits gender-specific clinical and biological characteristics, and considering these differences in diagnostic approaches is important for early diagnosis and effective treatment. In particular, evaluating female patients with lower diagnostic thresholds, taking into account atypical symptoms and the postmenopausal period, may contribute to preventing cardiopulmonary complications due to late diagnosis of the disease. These findings need to be confirmed by larger, multicenter studies.
